# Molecular Characterization and Function of Bone Morphogenetic Protein 7 (*BMP*7) in the Pacific Abalone, *Haliotis discus hannai*

**DOI:** 10.3390/genes14061128

**Published:** 2023-05-23

**Authors:** Jianfang Huang, Mingcan Zhou, Weiwei You, Xuan Luo, Caihuan Ke

**Affiliations:** 1 State Key Laboratory of Marine Environmental Science, College of Ocean and Earth Sciences, Xiamen University, Xiamen 361102, China; 2 Institute of Oceanography, College of Geography and Oceanography, Minjiang University, Fuzhou 350108, China; 3 State Key Laboratory of Mariculture Breeding, Xiamen University, Xiamen 361102, China

**Keywords:** BMP7, *Haliotis discus hannai*, growth, RNAi, SNP

## Abstract

Bone morphogenetic proteins (BMPs) play important roles in a lot of biological processes, such as bone development, cell proliferation, cell differentiation, growth, etc. However, the functions of abalone BMP genes are still unknown. This study aimed to better understand the characterization and biological function of BMP7 of *Haliotis discus hannai* (*hdh-BMP*7) via cloning and sequencing analysis. The coding sequence (CDS) length of hdh-BMP7 is 1251 bp, which encodes 416 amino acids including a signal peptide (1–28 aa), a transforming growth factor-β (TGF-β) propeptide (38–272 aa), and a mature TGF-β peptide (314–416 aa). The analysis of expression showed that *hdh-BMP*7 mRNA was widely expressed in all the examined tissues of *H. discus hannai*. Four SNPs were related to growth traits. The results of RNA interference (RNAi) showed that the mRNA expression levels of *hdh-BMPR* I, *hdh-BMPR* II, *hdh-smad*1, and *hdh-MHC* declined after *hdh-BMP*7 was silenced. After RNAi experiment for 30 days, the shell length, shell width, and total weight were found to be reduced in *H. discus hannai* (*p* < 0.05). The results of real-time quantitative reverse transcription PCR revealed that the *hdh-BMP*7 mRNA was lower in abalone of the S-DD-group than in the L-DD-group. Based on these data, we hypothesized that BMP7 gene has a positive role in the growth of *H. discus hannai*.

## 1. Introduction

The transforming growth factor β (TGF-β) family is a multifunctional group of secretory signal transducers that are involved in cell proliferation, differentiation, apoptosis, embryonic development, growth, bone formation and reconstruction, and other important biological processes [1,2,3,4,5]. The TGF-β superfamily includes TGF-βs, growth differentiation factors (GDFs), bone morphogenetic proteins (BMPs), Inhibins/Activins, and Nodal. All members of the TGF-β superfamily release mature polypeptides by division of precursors at specific sites, and the activity relies on the formation of dimers from the same or different subunits [6,7]. Although the TGF-β family contains many diverse factors, they all signal via conserved signaling pathways. The TGF-β family ligands bind to receptors and then mediate the phosphorylation of Smad proteins. The interaction between Smads and other signaling proteins mediates regulatory signals, controlling the expression of target genes, the translation of mRNA translation, and the regulation of cytoplasmic protein [8,9,10,11]. In recent years, the TGF-β superfamily has been found to play a critical role in the growth and development of shellfish [7,12,13,14].

BMPs, as the largest secreted cytokines in the TGF-β superfamily, are originally named for their ability in bone formation [15]. BMPs are widely expressed during the development of mammals, with many biological activities, such as development, proliferation, etc. [7,16]. BMPs can distinguish between serine and threonine kinase receptors and bind to them, and the subsequent signaling is mediated by Smad-dependent and -independent pathways [7,17,18]. BMP7, as a member of the BMPs, plays important roles in various biological functions including cell proliferation, differentiation, and apoptosis [19,20,21,22,23,24]. BMP7 has the classical TGF-β domains [7,25]. In vertebrates, BMP7 is closely related to bone development, organogenesis, and embryo development [20,22,23,26]. Recombinant human BMP7 protein can promote bone regeneration [7,27,28,29]. In mice, BMP7 was related to the proliferation of sertoli cells, and BMP7 knockout mice were infertile [7,30,31]. BMP7 was showed to be associated with growth in cattle and chickens, and growth-related SNPs have been founded [7,32,33,34]. Overexpression of BMP7 promotes muscle growth and prevents muscle atrophy [35,36]. In invertebrates, so far, the BMP7 gene has been reported in several bivalve species, including *Tegillarca granosa* [37], *Pinctada martensii* [38,39], and *Hyriopsis cumingii* [40]. The interference experiments of *PmBMP*7 showed that BMP7 played an important role in the formation of nymphal and columnar layers of shells in the *P. martensii* [38]. The mRNA expression of *TG-BMP*7 in the mantle and gill of *T. granosa* is high, and the expression level in the d-larva stage is high during embryo development, suggesting that BMP7 may be related to shell formation and regulation of embryo growth and development [7].

The *H. discus hannai* is the most popular cultivated abalone throughout in China [41,42]. The abalone growth cycle generally lasts for 2 years, which restricts the development of abalone aquaculture. At present, the molecular genetics and function of most genes closely associated with growth in *H. discus hannai* are not clear, which limits the genetic improvement of related growth traits. Hybridization is an effective method to introduce improved traits [43,44,45]. Lvpan abalone (*H. discus hannai♀× H. fulgens♂*), a hybrid with fast growth and large size, has become a new abalone variety in Fujian Province [46]. Lvpan abalone may be a good material for proving the mechanism related to abalone growth. BMP7 is identified to be involved in animal growth and development, but there are few studies conducted on BMP7 in marine mollusks, and there are no reports on whether BMP7 is involved in the growth of abalone. In this work, we analyzed the gene structure of BMP7 and studied its role in the *H. discus hannai* growth. Our results in the paper would lay a foundation for further study on molecular mechanisms of growth regulation in abalone.

## 2. Materials and Methods

### 2.1. Sample Collection

All abalones (*H. discus hannai* and Lvpan abalone) used in this study were obtained from Fuda Aquaculture and were consistent with the previous study [43]. The adductor muscles were collected in different stages of development of *H. discus hannai*, that is, at 1, 4, 7, 10, 12, 14, 16, and 18 months. All experimental samples used to analyze the expression pattern in tissues were consistent with the previous study [43]. The adductor muscle tissue samples of the larger *H. discus hannai* (“L-DD-group”), the smaller *H. discus hannai* (“S-DD-group”), the larger Lvpan abalone (“L-DF-group”), and the smaller Lvpan abalone (“S-DF-group”) were also consistent with the previous study [43]. All the samples were stored at −80 °C.

### 2.2. Cloning and Sequence Analysis

The isolation of total RNA and the synthesis of cDNA followed the methods in the previous study by Huang et al. (2023) [43]. The primers (*hdh-BMP*7-F and *hdh-BMP*7-R), used to amplify the *hdh-BMP*7 open reading frame (ORF), were designed using the software of Primer3Plus (Table 1). The ORF sequences of *hdh-BMP*7 were uploaded to GenBank (accession numbers: OP856631). Lasergene software tools were used to determine the amino acid sequence of hdh-BMP7. SingalP 5.0 Server was used for signal peptide prediction; Prop 1.0 server was used for proteolytic processing site analysis; SOPMA was used for secondary structure analysis; CDD was used for protein domains; the ClustalW2 program was used for aligning the sequence of BMP7, and the MEGA program with the neighbor-joining algorithm was used for constructing the phylogenetic tree.

### 2.3. Single Nucleotide Polymorphism Analysis

To explore the SNPs of *hdh-BMP7*, the abalones, the foot muscle samples, the method of DNA extraction, the growth-related traits, and the method of *hdh-BMP7* ORF sequence genotype were consistent with the previous study [43]. The POPGENE 1.32 software was used to evaluate the PIC of the SNPs according to the manufacturer’s instructions.

### 2.4. RNA Interference of hdh-BMP7

A pair of specific primers, *dsBMP*7-F and *dsBMP*7-R, were designed to amplify a 303 bp fragment of the *hdh-BMP*7. *EGFP* acted as a control gene, amplifying a 497 bp fragment. The information of specific primers is shown in Table 1. The dsRNA synthesis and purity were performed as previously described [47]. For the *hdh-BMP*7 RNAi assays, approximately 80 *H. discus hannai* (~4–5 g) were randomly divided into two groups: the *hdh-BMP*7 RNAi group and the *EGFP* control group. The abalones were intramuscularly injected. The RNAi experimental methods were consistent with the previous study [43].

### 2.5. Real-Time Quantitative Reverse Transcription PCR

The qRT-PCR was used to detect the gene expression levels of *hdh-BMP7* (gene ID: evm.model.scaffold76053.25.1), hdh-BMPR I (gene ID: evm.model.scaffold168485.18), hdh-BMPR II (gene ID: evm.model.scaffold36941.4), hdh-Smad1 (gene ID: evm.model.scaffold148433.104_evm.model.scaffold148433.105), and MHC (gene ID: evm.model.scaffold19291.11). These primers (Table 1) were designed using the software of Primer3Plus. The data processing used the 2^−∆∆CT^ method. The qRT-PCR runs and the cycling parameters used were performed using similar conditions as described previously [48]. The PCR amplification was performed in 20 μL reaction mixtures containing the following components: 10 μL FastStart Universal SYBR Green Master (ROX), 1 μL forward and reverse primers (10 μM each), 5 μL of cDNA (100-times diluted), and 4 μL distilled water [29].

### 2.6. Statistical Analysis

All experimental data in this study were analyzed using one-way ANOVAs (ANOVA) in SPSS 19.0 (IBM, Tulsa, OK, USA). The significance level for these analyses was specified at *p* < 0.05.

## 3. Results

### 3.1. Characterization of Hdh-BMP7

**Figure 1:**
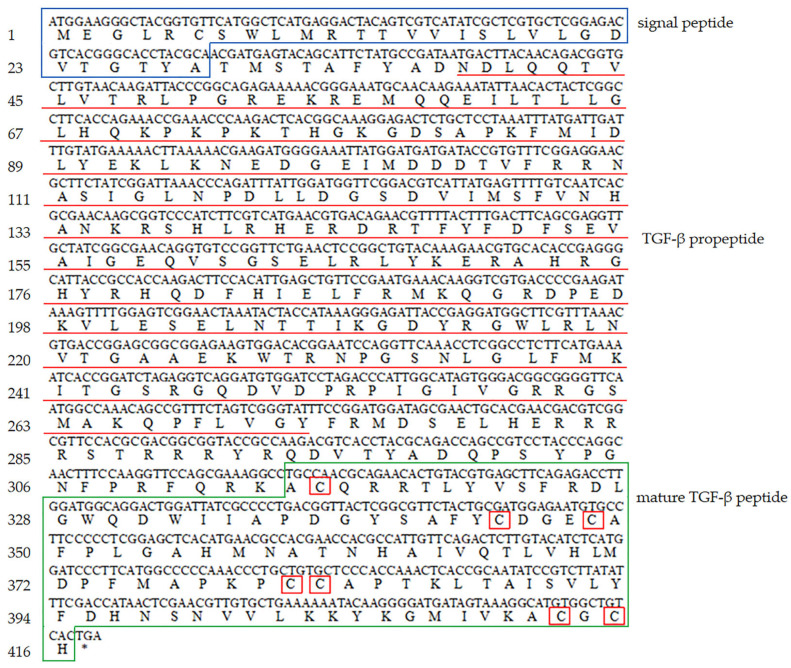
Gene information of *hdh-BMP7*. The cysteine residues are shown in the red boxes. The signal peptides are shown in the blue boxes. The TGF-β propeptide is shown in red lines. The mature TGF-β peptides are shown in the green boxes.

**Figure 2:**
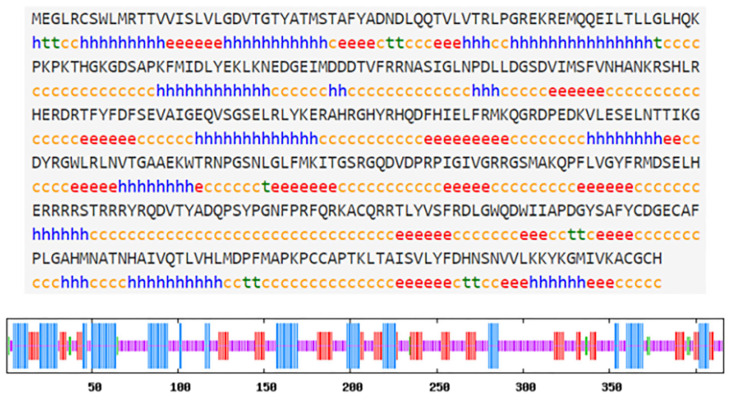
The information of hdh-BMP7 in secondary structure. Hh expresses the α-helix. Tt expresses the β-turn. Ee expresses the extended strand. Cc expresses the random coil.

**Figure 3:**
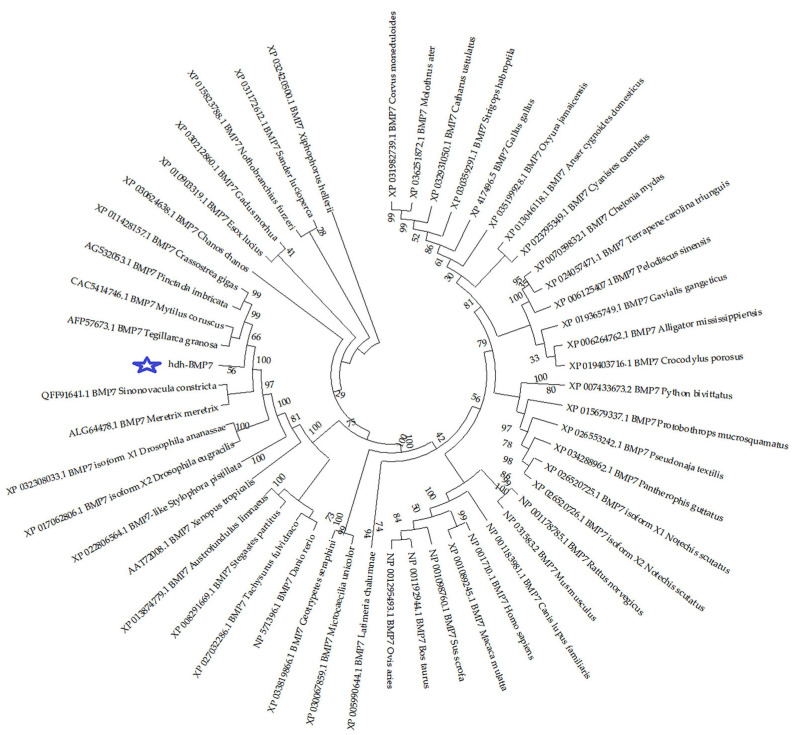
Phylogenetic tree of BMP7 from vertebrates and invertebrates. Hdh-BMP7 is shown with a pentagram.

The hdh-BMP7 protein contained 416 amino acid residues and was encoded by 1251 nucleotides. The estimated molecular weight of the mature hdh-BMP7 was 47.80 kDa, and the pl was 9.15. The hdh-BMP7 protein contained a signal peptide (1–28 aa), a TGF-β propeptide (38–272 aa), and a mature TGF-β peptide (314–416 aa). The mature protein has two putative proteolytic sites of Arg-X-X-Arg (52–55 aa and 282–285 aa) with seven conserved cysteine residues (Figure 1). The secondary structure of hdh-BMP7 was predicted to possess 26.44% α-helix, 20.43% extended strand, 2.88% β-turns, and 50.24% random coils (Figure 2). The phylogenetic tree demonstrated a close relationship between *H. discus hannai*, *T. granosa*, *Mytilus coruscus*, *Pinctada imbricata*, and *Crassostrea gigas*, which clustered together and formed a branch (Figure 3). This suggests that the BMP7 protein is evolutionarily conserved in sequence and structure in shellfish.

### 3.2. Expression Detection of Hdh-BMP7

**Figure 4:**
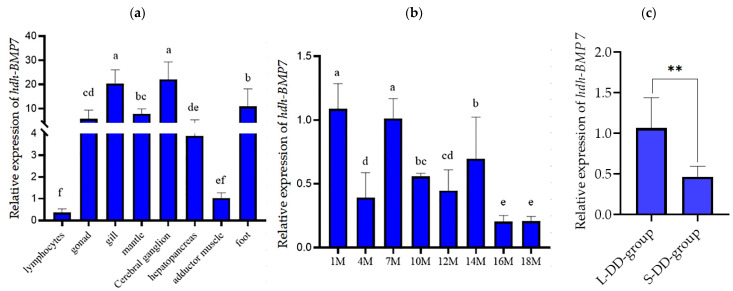
The expression pattern of *hdh-BMP*7 mRNA in (**a**) various tissues (*n* = 6), (**b**) development stages (*n* = 4), and (**c**) the L-DD-group (*n* = 12) and S-DD-group (*n* = 12). Different letters and ** indicate *p* < 0.05 and *p* < 0.01, respectively.

The expression levels of *hdh-BMP*7 in tissue and developmental stage-specific abalones were determined by qRT-PCR. The highest expression levels of *hdh-BMP*7 were found in the gills and cerebral ganglion (*p* < 0.05, Figure 4a). *Hdh-BMP*7 mRNA was widely expressed at different developmental stages of *H. discus hannai* (Figure 4b). The expression of *hdh-BMP*7 mRNA was lower in the S-DD-group than in the L-DD-group (*p* < 0.01, Figure 4c).

### 3.3. SNPs in Hdh-BMP7

Sequence comparisons detected eight single nucleotide polymorphisms (SNPs) in *hdh-BMP*7 (Table 2). The average Polymorphic information content (PIC) was 0.147, which is considered a low polymorphism level (PIC < 0.25). Four SNPs (744 A > G, 819 A > G, 834 T > A, and 852 C > G) were related to growth traits in *H. discus hannai*. The MW/TW of AA was higher than that of abalones with the genotype of GG at the 744 A > G locus (*p* < 0.05). The MW/TW of AA was higher than that of abalones with the GG and GA genotype at the 819 A > G locus (*p* < 0.05). The MW/TW of TT was lower than that of abalones with the AT genotype at the 834 T > A locus (*p* < 0.05). The MW/TW of CC was higher than that of abalones with the genotype of GG at the 852 C > G locus (*p* < 0.05).

### 3.4. Effects of RNA Interference

**Figure 5:**
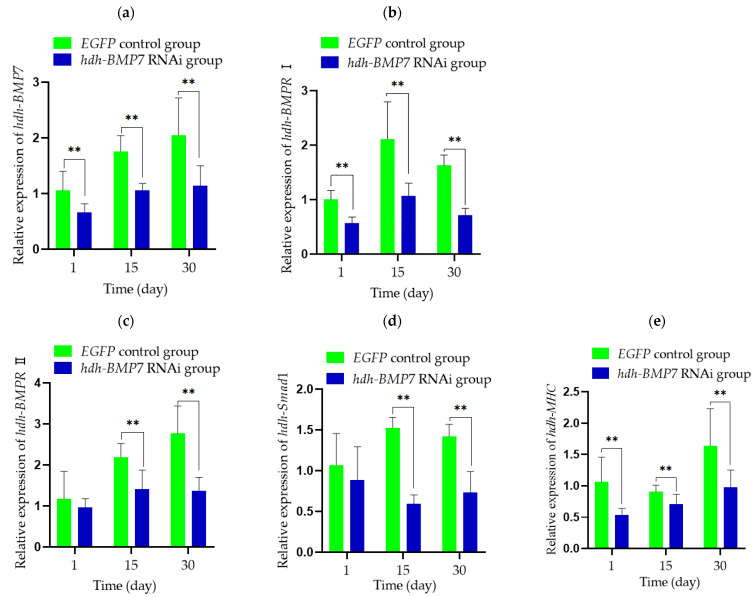
Expression analysis of RT-qPCR. Expression analysis of (**a**) *hdh-BMP*7, (**b**) *hdh-BMPR* I, (**c**) *hdh-BMPR* II, (**d**) *hdh-Smad*1, and (**e**) *hdh-MHC* after RNAi in adductor muscle (*n* = 4). ** indicate *p* < 0.05.

The expression levels of *hdh-BMP*7 in the adductor muscle decreased about 40–50% after RNAi experiment at 1 day, 15 days, and 30 days (*p* < 0.01, Figure 5a) compared to the *EGFP* control group. After RNAi experiment for 1 day, *hdh-BMPR* I and myosin heavy chain (*hdh-MHC*) mRNA levels were significantly lower in the *hdh-BMP*7 RNAi group than in the *EGFP* control group (Figure 5b,e). After RNAi experiment for 15 and 30 days, *hdh-BMPR* I, *hdh-BMPR* II, *hdh-Sma*d1, and *hdh-MHC* mRNA levels were lower in the *hdh-BMP*7 RNAi group compared with the *EGFP* control group (*p* < 0.01, Figure 5).

**Figure 6:**
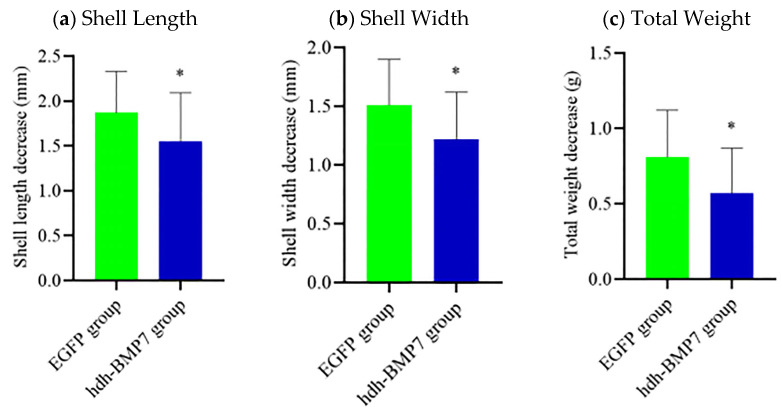
The growth characteristics after *hdh-BMP*7 RNAi in *H. discus hannai*. (**a**) The decrease in shell length after *hdh-BMP*7 RNAi. (**b**) The decrease in shell width after *hdh-BMP*7 RNAi. (**c**) The decrease in total weight after *hdh-BMP*7 RNAi. * represents *p* < 0.05.

After RNAi experiment for 30 days, the decrease in shell length, shell width, and total weight in the *hdh-BMP*7 RNAi group was significantly higher than in the *EGFP* control group (*p* < 0.05, Figure 6a–c).

### 3.5. Verification of the Lvpan Abalone

**Figure 7:**
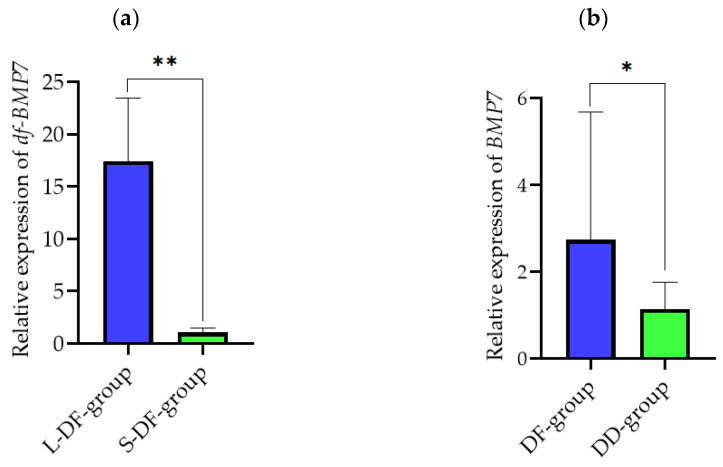
Expression of *BMP*7 mRNA in Lvpan abalone. (**a**) Expression of *hdh-BMP*7 in adductor muscle between L-DF-group (*n* = 12) and S-DF-group (*n* = 12). (**b**) Expression of *BMP*7 in adductor muscle between DD-group (*n* = 24) and DF-group (*n* = 24). * and ** indicate *p* < 0.05 and *p* < 0.01, respectively.

In order to further verify the function of BMP7 in abalone, we compared the expression level of BMP7 mRNA between *H. discus hannai* (DD) and Lvpan abalone (DF). The expression of *df-BMP*7 mRNA in Lvpan abalone was lower in the S-DF-group than in the L-DF-group (*p* < 0.05, Figure 7). In addition, the expression of *BMP*7 mRNA in the *H. discus hannai* (DD-group) was lower than in the Lvpan abalone (DF-group) (*p* < 0.05).

## 4. Discussion

BMP7 is one of the members of the TGF-β superfamily. In this paper, we cloned the ORF frame of the BMP7 from *H. discus hannai*. We found that the hdh-BMP7 protein contained 416 amino acid residues encoded by 1251 nucleotides. Our results also demonstrate that hdh-BMP7 has a TGF-β propeptide. The hdh-BMP7 protein has the same TGF-β functional domain as other BMP members, which is required for the BMP ligand to be functional [49]. Hdh-BMP7 has seven conserved cysteine residues, which is consistent with the BMP7 in *Meretrix meretrix* [50]. However, not all mollusks have seven highly conservative cysteine residues, such as *Sinonovacula constricta* [7] and *T. granosa* [37]. Highly conserved cysteine residues are the structural characteristics that are typical of the TGF-β superfamily. Therefore, the data indicate that hdh-BMP7 is a member of the BMP family [51]. The result of the phylogenetic tree suggests that *H. discus hannai* is most closely related to these shellfish of *T. granosa*, *M. coruscus*, *P. imbricata*, and *C. gigas*.

BMPs are widely expressed during the development of mammals, with many biological activities [7,16]. BMP7 is a member of the BMPs. To explore the potential roles of BMP7 in *H. discus hannai*, we analyzed the expression of *hdh-BMP*7 mRNA in various tissues of individuals in different development stages. The qRT-PCR results revealed that *hdh-BMP*7 was widely expressed in the cerebral ganglion, gills, mantle, and other tissues, which is consistent with results previously found in zebrafish [52], *M. meretrix* [50] and *T. granosa* [37]. Previous studies have suggested that the BMP system plays a role in pituitary function in vertebrates [53], and BMP7 is mainly involved in shell growth and nacre formation [7,37,50]. Thus, our results of tissues distribution suggest that *hdh-BMP*7 may be related to shell growth and shell formation and play an important role in the nervous system. In addition, the *hdh-BMP*7 mRNA was widely expressed in different developmental stages of abalone, and its expression level was higher in the L-DD-group than in the S-DD-group. Thus, we speculate that *hdh-BMP*7 may be related to the growth and development of *H. discus hannai*.

SNP is an important molecular marker, widely divided in the genome of organisms. The SNPs in candidate genes were analyzed to find the loci related to the target trait, which helps to effectively screen individuals. For example, in *Haliotis diversicolor supertexta*, a total of nine SNP sites were identified in the MSTN gene. The association analysis of SNP sites and growth traits showed that the SNP site g909C > T in the coding region of the MSTN gene in *H. diversicolor supertexta* was significantly correlated with the shell length, shell height, and body weight [54]. In the myostatin gene of *H. discus hannai*, nine SNPs were significantly associated with growth traits [43]. In previous studies, BMP7 has been identified as a potential growth-related gene in SNP studies in bovines [33] and chickens [34] but not in abalone. In order to explore the SNPs and growth correlation of the BMP7 gene of *H. discus hannai*, *hdh-BMP7* was experimentally measured by direct sequencing of PCR products, and the association between *hdh-BMP7* SNPs and five growth-related traits (shell length, shell width, total weight, muscle weight, and muscle weight/wet weight ratio) was analyzed. These data identified eight SNPs in the CDS region of *hdh-BMP*7, four of which were significantly associated with growth traits, which suggests that *hdh-BMP*7 is closely correlated with growth traits of abalone. Previous research has shown that BMP signaling is the dominant pathway controlling muscle mass and the inhibition of BMP signaling causes muscle atrophy [35]. Overexpression of BMP7 promotes muscle growth and prevents muscle atrophy [36]. In our study, the higher ratio of MW/TW means that the adductor muscle contributes to a large part of the total weight, which suggests that *hdh-BMP*7 also may be related to the abalone muscle growth. There is a strong correlation between BMP7 and the growth of shellfish such as *S. constricta* [7], *M. meretrix* [50], and *T. granosa* [37]. Therefore, we speculate *hdh-BMP*7 may play a crucial role in the process of growth regulation of *H. discus hannai*. In this study, only four SNP sites related to growth traits were found in the CDS region. In order to provide more useful molecular markers for breeding, we plan to screen SNP in the intron and promoter regions next. Meanwhile, we will further expand the abalone samples to verify the accuracy of SNP.

Although there are some reports on functional genes related to abalone growth, most of these studies use high-throughput sequencing technology to mine relevant genes and pathways and lack further functional verification, or the functional verification only stays at the level of cloning and mRNA expression, and the in-depth study on the mechanism of abalone functional gene is still very lacking. The technology of RNAi refers to the phenomenon of the gene silencing by foreign dsRNAs at the mRNA level. At present, researchers often use RNAi to reveal the genetic function of aquatic animals, such as *H. discus hannai* [43], *P. martensii* [38], etc. To further investigate the interaction between the BMP system and growth in abalone, we successfully inhibited the expression of *hdh-BMP*7 by RNAi, which resulted in a significant decrease in shell length growth gain, shell width growth gain, and total weight gain compared with the *EGFP* control group. This further demonstrates that *hdh-BMP*7 can promote abalone growth. In vertebrates, BMPs can act as regulators of myoblasts and facilitate the development and regeneration of myoblasts after muscle injury [55,56]. Overexpression of BMP7 or ALK3 promotes muscle growth and prevents muscle atrophy [35,36]. A previous study [57] suggested that BMP signaling is a positive regulator of muscle mass in animals. In this study, the ratio of MW/TW was significantly different in several SNP loci. Muscle is the main edible part of abalone, accounting for about half of the body weight. The growth of muscle directly affects the overall growth. Some studies have investigated the mechanism of BMP7 in growth regulation on shellfish [7,37,50], although no reports have been made about abalone. In a future study, we will conduct more long-term RNAi experiments and detect the morphological change of abalone muscle fibers, so as to further reveal the regulatory function of BMP7 on regulating abalone muscle growth.

As we all know, BMPs initially bind to two BMP receptors, BMPR I and BMPR II, which activate Smads and target genes [58]. The interaction between Smads and other proteins mediates signals, controlling the expression of target genes, the translation of mRNA translation, and the regulation of cytoplasmic protein [8,9,10,11]. Previous studies have reported that TGF-β signaling was transduced from the extra-cellular space to the cell nucleus via Smad proteins to activate downstream pathways and regulate general life processes [59,60]. In *Scylla paramamosain*, BMP7 also obeys this signaling mechanism [15]. In this study, when *hdh-BMP*7 expression was inhibited, the expression levels of *hdh-BMPR* I and *hdh-BMPR* II changed in response, which indicates that *hdh-BMPR* I and *hdh-BMPR* II are the receptors of *hdh-BMP*7 in abalone. In addition, the expression levels of *hdh-smad*1 and *hdh-MHC* were significantly decreased (*p* < 0.01) compared to the *EGFP* control group at 15 days and 30 days, suggesting that *hdh-BMP*7 may promote muscle growth by *hdh-Smad*1 signaling in *H. discus hannai*.

In the end, we verified the role of *BMP*7 in Lvpan abalone. Lvpan abalone, a hybrid with fast growth, has become a good material for proving the mechanism related to abalone growth. In this research, we compared the expression level of BMP7 mRNA between *H. discus hannai* (DD) and Lvpan abalone (DF). We found that the expression in L-group DD and DF at the same age was higher than that in S-group equivalents. The *BMP*7 expression level in the DF was higher than that in DD at the same age, which further indicates that *BMP*7 plays a positive role in regulating abalone growth. Ultimately, our data indicate that *hdh-BM*P7 potentially impacts the growth of *H. discus hannai*.

## 5. Conclusions

In the study, we cloned the ORF sequence and described the structure of *hdh-BMP*7 from *H. discus hannai*. The expression level of *hdh-BMP*7 mRNA was higher in the fast-growing group (*p* < 0.05) than in the slow-growing group, suggesting that it may be correlated with the growth of abalone. Further results from RNAi indicated that the *hdh-BMP*7 gene plays a vital role in promoting growth of *H. discus hannai*. Association analysis identified four SNPs that were significantly associated with growth traits, suggesting that *hdh-BMP*7 is closely involved in abalone growth. The results of this paper would lay a foundation for further study on the function of *hdh-BMP*7 in abalone growth and contribute to molecular marker assisted breeding in *H. discus hannai*.

## Figures and Tables

**Table 1:** Sequences of the primer pairs.

Primer Name	Sequence Information (5′-3′)
*hdh-BMP*7-F	CACAGAAGGGGATCGAGTCA
*hdh-BMP*7-R	AGGCTACGAAAAGGTCACGT
*hdh-BMP*7-dsF	TAATACGACTCACTATAGGGGCCACCAAGACTTCCACATT
*hdh-BMP*7-dsR	TAATACGACTCACTATAGGGGATCGCTATCCATCCGAAAA
*EGFP*-dsF	TAATACGACTCACTATAGGGGTGCCCATCCTGGTCGAGCT
*EGFP*-dsR	TAATACGACTCACTATAGGGTGCACGCTGCCGTCCTCGAT
*hdh-BMP*7-qF	GCGGTCCCATCTTCGTCA
*hdh-BMP*7-qR	TGCACGTTCTTTGTACAGCC
*hdh-BMPR* I-qF	GACCTCATAGAACAGTCCC
*hdh-BMPR* I-qR	ACCATAACGCCCCTTGCCT
*hdh-BMPR* II-qF	GGCGGGGAGAGATGTAATG
*hdh-BMPR* II-qR	TAAGTTGGGTCGGGTGTAG
*hdh-Smad*1-qF	GGACTCCTCTCCAACGTCAA
*hdh-Smad*1-qR	ACATTCCGCAAACACCTCAC
*hdh-MHC*-qF	GACCCCAACGACCCTGATAT
*hdh-MHC*-qR	TCTTCTCCCTTGGTGCTCTG
*β-actin*-qF	GGTATCCTCACCCTCAAGT
*β-actin*-qR	GGGTCATCTTTTCACGGTTG

**Table 2:** Growth traits of *H. discus hannai* with different genotypes.

Locus	Genotype	Sample Number	Shell Length (mm)	Shell Width (mm)	Total Weight (g)	Muscle Weight (g)	MW/TW
570 C > T	CT	111	73.76 ± 9.82 ^a^	49.64 ± 6.27 ^a^	42.07 ± 16.40 ^a^	17.48 ± 7.92 ^a^	0.4064 ± 0.0458 ^a^
	TT	104	73.99 ± 9.61 ^a^	49.86 ± 6.21 ^a^	41.25 ± 15.29 ^a^	16.73 ± 7.15 ^a^	0.3981 ± 0.0407 ^a^
	CC	4	71.19 ± 7.99 ^a^	48.03 ± 4.85 ^a^	36.63 ± 15.55 ^a^	15.30 ± 8.35 ^a^	0.4071 ± 0.0501 ^a^
606 C > T	CC	111	74.47 ± 9.60 ^a^	50.11 ± 6.13 ^a^	42.10 ± 15.57 ^a^	17.06 ± 7.30 ^a^	0.3975 ± 0.0416 ^a^
	TC	106	73.21 ± 9.48 ^a^	49.30 ± 6.14 ^a^	41.05 ± 15.75 ^a^	17.08 ± 7.68 ^a^	0.4066 ± 0.0451 ^a^
744 A > G	AA	139	73.67 ± 9.72 ^a^	49.66 ± 6.18 ^a^	42.55 ± 16.36 ^a^	17.62 ± 7.83 ^a^	0.4060 ± 0.0439 ^a^
	GA	74	74.30 ± 9.20 ^a^	49.88 ± 6.08 ^a^	40.15 ± 14.59 ^a^	16.33 ± 6.95 ^a^	0.3985 ± 0.0422 ^ab^
	GG	4	69.57 ± 16.74 ^a^	47.47 ± 10.31 ^a^	32.95 ± 15.75 ^a^	12.13 ± 6.51 ^a^	0.3595 ± 0.0385 ^b^
805 A > G	AG	107	73.44 ± 9.29 ^a^	49.50 ± 6.08 ^a^	41.53 ± 16.17 ^a^	17.20 ± 7.77 ^a^	0.4056 ± 0.0448 ^a^
	GG	106	74.07 ± 9.96 ^a^	49.88 ± 6.26 ^a^	41.47 ± 15.45 ^a^	16.91 ± 7.33 ^a^	0.3994 ± 0.0426 ^a^
	AA	5	75.21 ± 12.42 ^a^	49.81 ± 8.41 ^a^	41.84 ± 17.68 ^a^	16.80 ± 8.31 ^a^	0.3929 ± 0.0403 ^a^
819 A > G	AA	86	73.47 ± 9.41 ^a^	49.56 ± 5.95 ^a^	41.88 ± 16.24 ^a^	17.58 ± 7.83 ^a^	0.4115 ± 0.0433 ^a^
	GA	97	74.32 ± 9.69 ^a^	50.09 ± 6.39 ^a^	42.40 ± 15.87 ^a^	17.27 ± 7.56 ^a^	0.3987 ± 0.0448 ^bc^
	GG	35	73.10 ± 10.30 ^a^	48.94 ± 6.30 ^a^	38.14 ± 14.31 ^a^	15.14 ± 6.59 ^a^	0.3895 ± 0.0370 ^c^
834 T > A	TT	174	73.14 ± 9.74 ^a^	49.20 ± 6.24 ^a^	40.48 ± 15.74 ^a^	16.49 ± 7.47 ^a^	0.3988 ± 0.0445 ^a^
	AT	42	75.74 ± 8.59 ^a^	51.24 ± 5.50 ^a^	44.51 ± 14.95 ^a^	18.70 ± 7.15 ^a^	0.4144 ± 0.0367 ^b^
852 C > G	CC	133	72.93 ± 9.47 ^a^	49.33 ± 6.09 ^a^	41.33 ± 15.97 ^a^	17.12 ± 7.66 ^a^	0.4058 ± 0.0440 ^a^
	GC	80	75.28 ± 9.43 ^a^	50.29 ± 6.11 ^a^	41.97 ± 15.47 ^a^	17.06 ± 7.34 ^a^	0.3982 ± 0.0421 ^ab^
	GG	4	69.57 ± 16.74 ^a^	47.47 ± 10.31 ^a^	32.95 ± 15.75 ^a^	12.13 ± 6.51 ^a^	0.3595 ± 0.0385 ^b^
903 G > C	GG	124	73.08 ± 9.88 ^a^	49.36 ± 6.30 ^a^	41.80 ± 16.26 ^a^	17.39 ± 7.84 ^a^	0.4070 ± 0.0445 ^a^
	CG	86	74.83 ± 8.86 ^a^	50.17 ± 5.85 ^a^	40.82 ± 14.64 ^a^	16.53 ± 6.88 ^a^	0.3979 ± 0.0407 ^a^
	CC	7	72.16 ± 14.87 ^a^	48.48 ± 9.01 ^a^	42.29 ± 22.71 ^a^	16.69 ± 10.60 ^a^	0.3781 ± 0.0506 ^a^

Note: Different little letters within a column are *p* < 0.05. MW means muscle weight; TW means total weight.

## Data Availability

Not applicable.
